# Optimization of Aquatic-Terrestrial Ecosystem in Relation to Soil Nitrogen Status for the Cultivation of Fish and Aquatic Food Crops of the Indian Subtropics

**DOI:** 10.1100/tsw.2001.455

**Published:** 2001-12-08

**Authors:** A.M. Puste, D.K. Das

**Affiliations:** Department of Agronomy, Bidhan Chandra Krishi Viswavidyalaya, Nadia, West Bengal, India

**Keywords:** aquatic-terrestrial, ecosystem, nitrogen, water chestnut, makhana, fish

## Abstract

A case study was undertaken during wet and postwet seasons to improve the perennial and alternate submerged saucer-shaped ponded lands (tal and semi-tal lands) in the coasts and northeastern plains of the Indian subtropics through pisciculture and cultivation of starch- and protein-rich aquatic food crops like water chestnut (Trapa bispinosa Roxb.) and makhana or fox nut (Euryale ferox Salisb.). The study revealed that the physico-chemical properties of soils (pH, organic C, organic matter, available N, P, and K) as well as quality of water (pH, EC, BOD, COD, CO3+, HCO3-, NO3-N, SO4-S, and Cl-), growing fish, makhana, and water chestnut was remarkably influenced by different moisture regimes and exhibited a significant improvement of soil health. The amount of organic C, available N, P, and K content were found significantly highest in the treatment where makhana was grown under alternate flooding and drying situation with a depth >2 m as compared to other treatments. Such enrichment of soil fertility, particularly in available N and P content, might be due to the accumulation of considerable amounts of biomass and fish excreta and their subsequent decomposition in situ in the soils. Therefore, the present study suggests that the N-enriched soil may effectively be utilized further for growing subsequent arable crops surroundings during summer season, which not only saves the amount of applied N fertilizer but also increases the apparent N efficiency with simultaneous increase in yield, and would benefit the farmers in this region.

## INTRODUCTION

The northeastern part of India has chains of rivers, intersected with many tributaries and canals. This has made possible a saucer-shaped wetland ecosystem bounded by land. Categorically, wetlands are lands transitional between terrestrial and wet areas, where the soil is frequently waterlogged during rainy months (either permanently, semipermanently, or temporarily), the water table is usually at or near the surface, or the land is inundated by varying depths of water.

Wetland comprises 6.4% (8.558 million km$^2$) of the world's total land area[1], of which 23.5 million ha is in India[2], mostly in the northeastern part of the country. The survival of human civilization is inextricably linked with wetlands, which sustain the economic stability of hundreds of million of people. And this swampy environment of the carboniferous period produced and preserved many of the fossil fuels on which we greatly depend now. Thus James[3] has rightly termed these areas as "Nature's kidney."

Besides the production of food (deep-water rice, fish, water chestnut, makhana, water lily, *Colocasia* spp., etc.) and nonfood crops (*Cyperus* spp., *Typha* spp., *Clinogyne dichotoma*, *Aeschynomene aspera*, *Brachiaria mutica*, *Coix* spp., etc.), wetlands are continuously enriched by the addition of large quantities of biomass, and the soil is enriched in consequence. Makhana or gorgon nut (*Euryale ferox* Salisb.) under the family Nymphaceae, and water chestnut (*Trapa bispinosa* Roxb.) under the family Trapaceae or Onagraceae, are annual floating-leaved herbs (with $C_3$ type of photosynthesis) and important aquatic food crops, growing in diverse areas from the tropics to the frigid zones, with a great importance to a wide sector of rural people. They are native to Southeast Asia, and prevalent in tropical and subtropical regions with humid to subhumid environments, like China, Japan, Malaysia, Thailand, the Philippines, Java, Sumatra, Nepal, Bangladesh, Sri Lanka, and India.

The water chestnut has often been used in studies on biomass production[4,5] and favourability for ecosystems[6,7,8,9]. Likewise, the biomass yield of makhana, growing in ponds in India, was positively correlated with N, K, and organic C, with the electrical conductivity of the soil, and with the contents of N, K, P, Na, Cl, $HCO_3^-$, Ca + Mg, and $SO_4^{2-}$[10,11]. The introduction of fish in waste wetland ecosystems for the utilization for food as well as for improving soil fertility by grazing on aquatic biomass and contributing through their faeces to N accumulation at soil surface has also been studied[12,13]. A typical grayish-black– to black-coloured soil dominated by clay (mucky type) is the main characteristic feature in low-lying areas of this zone. Indeed, organic matter is comparatively high, but, due to anaerobic condition prevailing for many months during wet season, it is partially decomposed. The soil status may further be improved if some period is allowed for quick decaying of such waste materials during postwet months under aerobic conditions. In this region, one of the most conventional practice by farmers is to utilize this resource-rich humus soil for the succeeding arable crops. This practice not only saves a substantial amount of fertilizer N including other important essential elements but also improves the physical condition of the soil.

From the literature survey, it is apparent that currently very limited or no information is available in the study zone or our country regarding the evaluation of soil fertility relating to soil N under integrated aqua-terrestrial ecosystem. To realize the facts with great importance, a case study was undertaken during wet and postwet seasons to improve the soil nutrient status of perennial and alternate submergence (referred to here as *tal* and *semi-tal* lands) in the coast and northeastern part of the Indian subtropics through pisciculture and the cultivation of water chestnut and makhana.

## MATERIALS AND METHODS

The field case study was undertaken during wet and postwet season at low-lying situations (*tal*, *semi-tal*, and temporary in nature) at Bidhan Chandra Krishi Viswavidyalaya, in Mohanpur (West Bengal), India, situated at 23° 5′ N latitude and 89° 0′ ’ E longitude and elevated at 9.75 m above mean sea level. The field investigation was carried out experimentally in a split-plot design, where treatments like cultivation of pisciculture and two important aquatic food crops (water chestnut and makhana) were considered as main plot treatments ($F_1$: fish; $F_2$: makhana; $F_3$; water chestnut) and varying depth of wet situations were considered as subplot treatments of the experiment, viz.:

- $T_1$: Continuous flooding (permanent *tal* lands) of 2.5 ± 0.5 m of water depth
- $T_2$: Continuous flooding (permanent in nature) of 1.5 ± 0.5 m of water depth
- $T_3$: Flooding during rainy and drying during summer season (semipermanent) of >2.0 m of water depth
- $T_4$: Flooding during rainy and drying during summer season (semipermanent) of 1.5 to 2.0 m of water depth
- $T_5$: Flooding during rainy and drying during winter including part summer (temporary) of =1 m of water depth

Initial soil samples were collected from the respective ponds for determination of physico-chemical characteristics like pH (6.76), organic C (0.46%), organic matter (0.84%), ammoniacal N (0.045%), nitrate N (0.024%), available N (0.067%), P (65.0 kg $ha^{-1}$), and K (274.6 kg $ha^{-1}$). Postexperimental soil samples for such characteristics were also determined. Water samples were collected for analyzing, initially for pH (7.01), BOD (1.59 µg $l^{-1}$), COD (2.28 µg $l^{-1}$), $CO_3^-$ (1.24 meq $l^{-1}$), $HCO_3^-$ (1.62 meq $l^{-1}$), $NO_3$-N (27.16 µg $l^{-1}$), $SO_4$-S (504.71 µg $l^{-1}$), and $Cl^-$ (174.32 µg $l^{-1}$), and finally, by AAS following the standard analytical procedure[14]. Electrical conductivity of such water samples was analyzed by Wheatston Conductivity Bridge following the standard method described by Jackson[14].

Due to their heterogeneous characters, fish and crops were compared by fish equivalence in terms of production, which was determined following this formula:

$$\text{Fish yield equivalent (t ha}^{-1}\text{)} = \frac{\text{Total price of the crop to be compared (Rs.)}}{\text{Price of fish t}^{-1}\text{ (Rs.)}}$$

For the calculation of fish yield equivalent and monetary returns, price of the products were considered as shown in Table 1.

## RESULTS AND DISCUSSION

### Soil Characteristics

#### *Food Crops*

Physical properties of pond bottom soils were significantly improved due to the cultivation of aquatic food crops (including

**TABLE 1**
**Fish Yield Equivalents and Monetary Returns**

| Food Crops | Price (Rs. $t^{-1}$) | Price (U.S.$ $t^{-1}$) |
|---|---|---|
| Fish | 45,000 | 957.40 |
| Makhana | 30,000 | 638.30 |
| Water chestnut | 6,000 | 127.70 |

*Note*: U.S.$1 = Indian Rs. 47.

the large amount of biomass added by fish culture) and greatly influenced by the different water depths maintained in wet areas (Table 2). The changes in soil pH varied from 6.62 to 7.64 in fish- and makhana-cultivated ponds; pH was 7.22 in water-chestnut–cultivated ponds. The changes of organic C, organic matter, $NH_4$-N, $NO_3$-N, total N, and available P and K content in soils varied from 0.51–0.74%, 0.89–1.28%, 0.048–0.076%, 0.036–0.048%, 0.084–0.124%, 72.4–118.6 kg $ha^{-1}$, and 292.5–413.8 kg $ha^{-1}$, respectively, in fish, water chestnut, and makhana ponds. Categorically, the highest values for all parameters were in makhana-cultivated plots, which may be due to the accumulation of relatively higher amount of organic matter causing higher available N, P, and K status in soils. Water chestnut plots had the next highest values.

### *Water Regimes*

Water regimes of varying depths also greatly influenced significantly the soil characteristics of the pond bottom due to the cultivation of makhana, water chestnut, and fish (Table 2). As regards to variation in water regime, it was observed that treatment $T_3$, where there was flooding during the rainy season to a depth of >2 m and drying during summer season, exhibited a higher amount of organic matter as well as available N, P, and K content in soils as compared to other water regimes. Such flooding during the rainy season and drying during summer season favours the release of available nutrients in soil due to the prevalence of partial anaerobic and aerobic situations *in situ* in the soil.

## Water Characteristics

### *Food Crops*

Water quality is an important criterion for habitats of aquatic flora and fauna and it was favourably reflected in this study. The results reveal that the changes in water quality varied with treatments (Table 3). However, the changes in water pH, EC, BOD, COD, $CO^{=}_3$, $HCO_3^-$, $NO_3$-N, $SO_4$-S, and $Cl^-$ concentration varied from 7.28–7.38, 0.69–0.78 $dSm^{-1}$, 1.49–2.36, 2.36–2.96, 1.44–1.76, 1.58–2.24, 30.48–32.55, 398.5–776.4, and 174.32–184.48 mg $l^{-1}$, respectively, during fish, makhana, and water chestnut cultivation.

### *Water Regimes*

In regard to the water characteristics of this case study it may be visualized that the corresponding values of pH, EC, BOD, COD, $CO_3^=$, $HCO_3^-$, $NO_3$-N, $SO_4$-S and $Cl^-$ concentration were varied from 6.98–7.37, 0.38–0.56 $dSm^{-1}$, 1.66–2.26, 2.31–2.99, 1.12–1.58, 1.57–2.23, 26.41–30.46, 502.5–635.3, and 168.71–196.68 mg $l^{-1}$, respectively, in different treatments of water regimes. The results show that the quality of water was much affected due to increased values of BOD (2.36 mg $l^{-1}$), COD (2.96 mg $l^{-1}$), $CO_3^=$ (1.76 mg $l^{-1}$), $HCO_3^-$ (2.24 mg $l^{-1}$), $SO_4$-S (776.4 mg $l^{-1}$), and $Cl^-$ (184.48 mg $l^{-1}$) in the treatment where pisciculture was practiced, while all these corresponding values excepting $HCO_3^-$ and $Cl^-$ content were much higher in treatment $T_5$ (flooding during the rainy season and drying during winter including part of summer of 1 m of water depth). Such changes in water quality due to fish cultivation may be attributed to higher rate of respiration causing greater depletion of oxygen and subsequently accumulation of $CO_2$ in the aquatic bodies.

## Crop Yield, Fish Yield Equivalent, and Monetary Return

### *Food Crops*

The results show that the yield of fish, makhana, and water chestnut were recorded as 1.43, 2.65, and 7.85 t $ha^{-1}$, respectively

**TABLE 2**
**Soil Fertility Status as Influenced by Pisciculture and Aquatic Food Crops Under Varying Water Regimes**

| Treatment | pH (Soil:Water 1:2.5) | Organic C (%) | Organic Matter (%) | Available N (%) | | Total N (%) | Available P (kg $ha^{-1}$) | Available K (kg $ha^{-1}$) |
|---|---|---|---|---|---|---|---|---|
| | | | | $NH_4^+N$ | $NO_3$-N | | | |
| **Food crops** | | | | | | | | |
| Fish | 6.62 | 0.51 | 0.89 | 0.048 | 0.036 | 0.082 | 72.4 | 292.5 |
| Makhana | 7.64 | 0.74 | 1.28 | 0.076 | 0.048 | 0.120 | 118.6 | 413.8 |
| Water chestnut | 7.22 | 0.63 | 1.14 | 0.068 | 0.042 | 0.112 | 86.3 | 378.6 |
| CD ($p$ = 0.05) | 0.45 | 0.14 | 0.16 | 0.006 | 0.008 | 0.012 | 27.40 | 31.8 |
| **Water regimes** | | | | | | | | |
| $T_1$ | 6.73 | 0.58 | 1.06 | 0.052 | 0.036 | 0.086 | 83.9 | 334.4 |
| $T_2$ | 6.99 | 0.61 | 1.14 | 0.057 | 0.035 | 0.090 | 92.8 | 363.4 |
| $T_3$ | 7.10 | 0.77 | 1.36 | 0.072 | 0.048 | 0.121 | 106.7 | 415.5 |
| $T_4$ | 7.69 | 0.68 | 1.31 | 0.062 | 0.052 | 0.114 | 99.3 | 384.8 |
| $T_5$ | 7.28 | 0.49 | 0.86 | 0.058 | 0.054 | 0.113 | 89.4 | 310.1 |
| CD ($p$ = 0.05) | 0.62 | 0.15 | 0.19 | 0.013 | 0.016 | 0.023 | 12.7 | 44.6 |

**TABLE 3**
**Water Quality as Influenced by Pisciculture and Aquatic Food Crops under Varying Water Regimes**

| Treatment | pH (Soil:Water 1:2.5) | EC ($dsm^{-1}$) | BOD ($\mu g\ l^{-1}$) | COD ($\mu g\ l^{-1}$) | $CO_3^{=}$ ($meq\ l^{-1}$) | $HCO_3^{-}$ ($meq\ l^{-1}$) | $NO_3^{-}N$ ($\mu g\ l^{-1}$) | $SO_4^{-}S$ ($\mu g\ l^{-1}$) | $Cl^{-}$ ($\mu g\ l^{-1}$) |
|---|---|---|---|---|---|---|---|---|---|
| **Food crops** | | | | | | | | | |
| Fish | 7.28 | 0.78 | 2.36 | 2.96 | 1.76 | 2.24 | 30.48 | 776.4 | 184.48 |
| Makhana | 7.32 | 0.69 | 2.02 | 2.36 | 1.49 | 1.86 | 32.55 | 562.6 | 174.32 |
| Water chestnut | 7.38 | 0.74 | 1.49 | 2.67 | 1.44 | 1.58 | 31.27 | 398.5 | 176.44 |
| CD ($p$ = 0.05) | N.S. | N.S. | 0.73 | 0.47 | 0.29 | 0.49 | 1.68 | 149.2 | 7.20 |
| **Water regimes** | | | | | | | | | |
| $T_1$ | 6.98 | 0.42 | 1.66 | 2.31 | 1.12 | 2.23 | 26.41 | 502.5 | 171.39 |
| $T_2$ | 7.12 | 0.38 | 1.82 | 2.44 | 1.34 | 1.99 | 28.22 | 556.2 | 168.71 |
| $T_3$ | 7.37 | 0.48 | 1.96 | 2.68 | 1.48 | 1.57 | 29.12 | 584.3 | 196.68 |
| $T_4$ | 7.35 | 0.51 | 2.09 | 2.88 | 1.39 | 1.81 | 27.92 | 617.6 | 182.96 |
| $T_5$ | 7.23 | 0.56 | 2.26 | 2.99 | 1.58 | 1.85 | 30.46 | 635.3 | 188.74 |
| CD ($p$ = 0.05) | 0.11 | 0.14 | 0.16 | 0.27 | 0.19 | 0.47 | 1.78 | 64.5 | 21.42 |

*Note:* N.S. = Not significant.

(Table 4). These yields are not comparable among themselves, due to their heterogeneous characters. However, the yield differences among the three were more pronounced when converted to equivalent yields of fish in respect to water regimes. Highest fish yield were obtained with $T_1$ (continuous flooding; permanent *tal* lands, of 2.5 ± 0.5 m of water depth), followed by makhana in $T_4$ (flooding during the rainy season and drying during the summer season, semipermanent >2.0 m of water depth) and water chestnut in $T_3$ (flooding during the rainy season and drying during summer season, semipermanent >2.0 m of water depth). Among these, makhana produced the highest fish equivalent and the increment was to the tune of 27.3 and 78.0% over fish and water chestnut, respectively.

**TABLE 4**
**Fish, Nut Yield, Fish Yield Equivalent, and Monetary Return of the Systems**

| Treatment | Yield ($t\ ha^{-1}$) | | | Fish Yield Equivalent ($t\ ha^{-1}$) | | | Gross Monetary Returns (Rs. $ha^{-1}$) | | |
|---|---|---|---|---|---|---|---|---|---|
| | Fish | Makhana | Water Chestnut | Fish | Makhana | Water Chestnut | Fish | Makhana | Water Chestnut |
| **Food crops** | | | | | | | | | |
| Fish | 1.43 | — | — | | | | | | |
| Makhana | — | 2.65 | — | | | | | | |
| Water chestnut | — | — | 7.89 | | | | | | |
| **Water regimes** | | | | | | | | | |
| $T_1$ | 1.72 | 2.24 | 7.56 | 1.72 | 1.49 | 1.01 | 77,400 (1,646.80) | 67,200 (1,429.80) | 45,360 (965.10) |
| $T_2$ | 1.37 | 2.82 | 8.48 | 1.37 | 1.88 | 1.13 | 61,650 (1,311.70) | 84,600 (1,800) | 50,880 (1,082.60) |
| $T_3$ | 1.67 | 2.96 | 9.23 | 1.67 | 1.97 | 1.23 | 75,150 (1,598.90) | 88,800 (1,889.40) | 55,380 (1,178.30) |
| $T_4$ | 1.42 | 3.28 | 9.12 | 1.42 | 2.19 | 1.22 | 63,900 (1,359.60) | 98,400 (2,093.60) | 54,720 (1,164.20) |
| $T_5$ | 0.96 | 1.95 | 5.05 | 0.96 | 1.30 | 0.67 | 43,200 (919.20) | 58,500 (1,244.70) | 30,300 (644.70) |
| CD ($P$ = 0.05) | 0.21 | 0.31 | 1.06 | — | — | — | — | — | — |

*Note*: Parentheses indicates values in U.S.$ (U.S.$1 = Indian Rs.47).

### Water Regimes

However, from the economic analysis it is found that the gross monetary return was recorded highest in makhana (Rs.98,400, which is equivalent to U.S.$2,093.60), particularly in treatment $T_4$, where the crop was raised in flooding conditions during the rainy season and drying during the summer season, with semi-permanent 1.5- to 2.0-m water depth of the pond.

Comparing the results of water regimes, it was found that the fish yield was highest (1.72 t ha$^{-1}$) in treatment $T_1$, where continuous flooding to a depth of $2.5 \pm 0.5$ m of water was maintained, whereas the highest yields of makhana (3.28 t ha$^{-1}$) and water chestnut (9.23 t ha$^{-1}$) were recorded in $T_4$ (flooding during the rainy season and drying during the summer season, semipermanent 1.5 to 2.0 m of water depth) and $T_3$ (flooding during the rainy season and drying during the summer season, semipermanent >2.0 m of water depth), respectively. Further, the results show that the fish yield equivalence was highest (2.19 t ha$^{-1}$) in treatment $T_4$, where makhana was cultivated under flooding during rainy season to a depth of 1.5–2.0 m and drying during summer months. However, the fish yield equivalence was recorded significantly lower in all the water regimes, particularly where water chestnut was cultivated.

## ACKNOWLEDGEMENTS

The authors expresses their sincere indebtedness and gratitude to the Indian Council of Agricultural Research of the Government of India for providing financial help for conducting field experiments.

| | | |
|---|---|---|
| Received: | July | 10, 2001 |
| Revised: | November | 12, 2001 |
| Accepted: | November | 15, 2001 |
| Published: | December | 8, 2001 |
